# Progressão da Lesão Miocárdica após Ablação por Radiofrequência em Crianças em Idade Escolar

**DOI:** 10.36660/abc.20220727

**Published:** 2024-01-24

**Authors:** Sissy Lara de Melo, Alberto Pereira Ferraz, Stephanie Ondracek Lemouche, Marcela Santana Devido, Gabriela Liberato de Sousa, Carlos E. Rochitte, Cristiano Faria Pisani, Denise Tessariol Hachul, Mauricio Scanavacca

**Affiliations:** 1 Hospital das Clínicas Faculdade de Medicina Universidade de São Paulo São Paulo SP Brasil Instituto do Coração do Hospital das Clínicas da Faculdade de Medicina da Universidade de São Paulo , São Paulo , SP – Brasil

**Keywords:** Ablação por Cateter, Contusões Miocárdicas, Criança, Segurança

## Abstract

**Fundamento:**

As últimas décadas têm assistido ao rápido desenvolvimento do tratamento invasivo de arritmias por procedimentos de ablação por cateter. Apesar da sua segurança e eficácia bem estabelecida em adultos, até o momento, há poucos dados nos cenários pediátricos. Uma das principais preocupações é a possível expansão da cicatriz do procedimento de ablação nessa população e suas consequências ao longo dos anos.

**Objetivos:**

Este estudo teve como objetivo analisar o risco da progressão da lesão miocárdica após ablação por cateter de radiofrequência em pacientes pediátricos.

**Métodos:**

Este é um estudo retrospectivo de 20 pacientes pediátricos com tratamento prévio de arritmia supraventricular com ablação, submetidos à ressonância magnética cardíaca e angiografia coronária para avaliação de fibrose miocárdica e da integridade das artérias coronárias durante o acompanhamento.

**Resultados:**

A idade mediana no procedimento de ablação foi 15,1 anos (Q1 12,9, Q3 16,6) e 21 anos (Q1 20, Q3 23) quando a ressonância magnética cardíaca foi realizada. Quatorze dos pacientes eram mulheres. Taquicardia por reentrada nodal e síndrome de Wolf-Parkinson-White foram os principais diagnósticos (19 pacientes), com um paciente com taquicardia atrial. Três pacientes apresentaram fibrose miocárdica ventricular, mas com um volume inferior a 0,6 cm ^3^ . Nenhum deles desenvolveu disfunção ventricular e nenhum paciente apresentou lesões coronarianos na angiografia.

**Conclusão:**

A ablação por cateter de radiofrequência não mostrou aumentar o risco de progressão de lesão miocárdica ou de lesões na artéria coronária.

**Figura Central:**
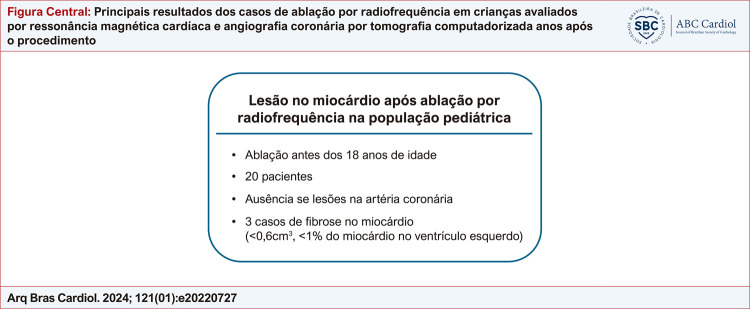
: Principais resultados dos casos de ablação por radiofrequência em crianças avaliados por ressonância magnética cardíaca e angiografia coronária por tomografia computadorizada anos após o procedimento

## Introdução

A eficácia e a segurança do tratamento das arritmias por ablação por cateter de radiofrequência (RF) está bem estabelecida na prática clínica atual. ^1 , 2^ Na população pediátrica acima de cinco anos de idade e peso maior que 15Kg, a conduta se tornou procedimento de rotina nos últimos anos. ^3^ Porém, existem poucos estudos e falta de evidência sobre o possível impacto da aplicação da RF no desenvolvimento do miocárdio em crianças na idade escolar e adolescentes. A maior parte do que se pratica hoje são inferências de estudos com animais. ^4 , 5^ As principais preocupações incluem o possível envolvimento das artérias coronárias e a progressão da fibrose no miocárdio durante a fase adulta. Existem poucos dados na literatura que nos permitem estimar o comportamento dessas lesões durante o crescimento do miocárdio, se elas tendem a aumentar ou não ao longo dos anos e quais seriam as consequências no desenvolvimento cardíaco da criança.

A maioria dos estudos na literatura demonstrou as consequências em curto prazo, desde horas até a alguns meses após a aplicação de RF. Nosso estudo teve como objetivo identificar a presença de lesões na artéria coronária e a extensão da fibrose no miocárdio após a aplicação de RF em pacientes pediátricos durante um acompanhamento de longo prazo.

## Métodos

Todos os 187 pacientes que foram submetidos à ablação por cateter com idade igual ou inferior a 15 anos em um centro terciário de cardiologia e que apresentavam 18 anos ou mais no início do estudo (2015) foram contatados e convidados para participar. Após o convite, 20 pacientes aceitaram a participar e um consentimento informado foi obtido dos pais ou representantes legais. Os protocolos de pesquisa foram revisados e aprovados (protocolo número SDC 3776/12/032).

Os procedimentos de ablação por RF foram realizados entre julho de 2002 e novembro de 2012. Por meio de punção da veia femoral direita e/ou esquerda e sob anestesia geral, cateteres de eletrodos multipolares foram introduzidos guiados por fluoroscopia para mapeamento eletrofisiológico e aplicação de pulsos de RF. A energia da RF foi aplicada usando cateteres de 4mm na maioria dos casos, exceto um paciente com taquicardia de Mahaim, em que um cateter com ponta de 8mm foi necessário após falha da ablação com um cateter com ponta de 4mm. A temperatura foi estabelecida em 60 ^o^ C e potência de 50W foi usada em todos os casos.

Para avaliação de fibrose miocárdica, ressonância magnética cardíaca (RMC) foi realizada, e a angiografia coronária por tomografia computadorizada (AngioTC) foi usada para investigação da integridade da artéria coronária. Todos os exames foram conduzidos com o equipamento Philips Achieva 1.5T MRI (Holanda). A função e os volumes ventriculares foram avaliados por cine-ressonância usando a sequência de pulsos de precessão livre no estado estacionário (SSFP, *Steady State Free Precession* ), e obtidos pelo método de Simpson com imagens no eixo curto do coração. Fibrose miocárdica foi avaliada usando a técnica do realce tardio do miocárdio, também conhecida por realce tardio pelo gadolínio (RTG), em aquisições em 2D e em 3D, avaliando os átrios e os ventrículos. Todas as análises foram realizadas usando o programa CVi42 (Circle CVi, Calgary, Canadá). A AngioTC foi realizada com um tomógrafo 320 x 0.5mm (Aquilion ONE, Canon Medical Systems, Otawara, Japão). O protocolo incluiu a aquisição de imagens sem contraste para o escore de cálcio e a aquisição com AngioTC com injeção de contraste iodado (50-70mL) e sincronização eletrocardiográfica prospectiva para avaliação do lúmen da artéria coronária.

### Análise estatística

Foi realizada uma análise descritiva. Os dados numéricos são apresentados em média e desvio padrão ou mediana e intervalo interquartil, de acordo com a normalidade dos dados. Os dados categóricos são apresentados em frequência absoluta e porcentagens. A distribuição dos dados foi testada quanto à normalidade pelo teste de Shapiro-Wilk. Um valor de p<0,05 foi considerado estatisticamente significativo. O programa IBM-SPSS para Windows, versão 25.0, foi usado para as análises, e o programa Microsoft Excel 2013 usado para tabular os dados.

## Resultados

Vinte pacientes foram submetidos à AngioTC e à RMC. A maioria dos pacientes (70%) era do sexo feminino, idade mediana de 15,1 (Q1: 12,9; Q3: 16,67) anos na ocasião da ablação e 20,8 (Q1: 20 e Q3: 23,5) anos no momento da avaliação. O período mediano entre a ablação e a avaliação foi 6,7 (Q1: 5,37; Q3: 9,12) anos. Peso médio dos participantes foi 65,7 ± 9Kg, altura 167,6±7,5cm e índice de massa corporal 23,4±2,3Kg/m ^2^ . A fração de ejeção média foi 61±8% e somente um paciente diagnosticado com taquicardia atrial (TA) apresentava doença cardíaca estrutural: um defeito septal atrial, corrigido cirurgicamente por atriosseptoplastia com colocação do retalho.

**Figura 1:**
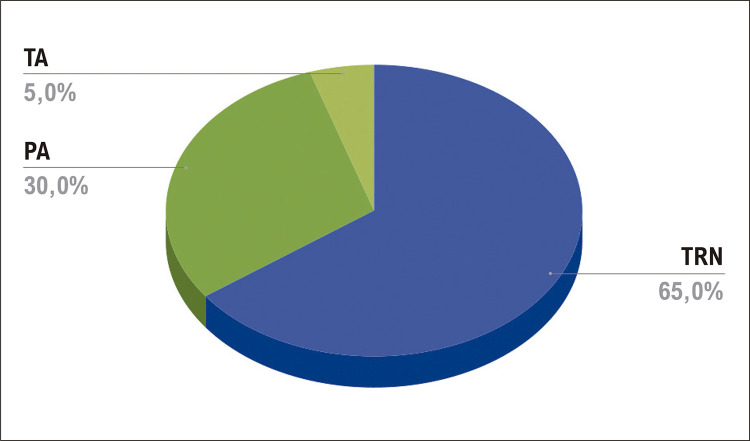
– Diagnóstico eletrofisiológico; VA: via acessória; TRN: taquicardia por reentrada nodal; TA: taquicardia atrial.

Dezoito pacientes foram submetidos à ablação na região septal direita: 13 com taquicardia por reentrada nodal e quatro com síndrome de Wolff-Parkinson-White (WPW). Os locais dessas vias acessórias (VAs) foram anterosseptal (n=1) e posterosseptal (n=3); um paciente apresentava TA localizada na região posterosseptal ( Tabela 1 , Figuras 1 e 2).

**Tabela 1:** – Características dos pacientes e dos procedimentos realizados

Paciente	Idade na ablação (anos)	Idade na RMC (anos)	Tempo para a RMC após a ablação (anos)	Diagnóstico	Local da ablação	Fibrose AD	Fibrose AE	Fibrose ventricular
1	12,3	20,6	8,3	TRN	AD (posterosseptal)			
2	14,3	20,5	6,2	VA	AD (posterosseptal)			
3	17,2	23,4	6,2	TRN	AD (posterosseptal)	X	X	
4	16,8	26,2	9,4	VA	AD (posterosseptal)		X	X
5	16,3	27,5	11,2	TRN	AD (posterosseptal)		X	
6	15,0	21,5	6,5	VA	AD (posterosseptal)			
7	17,6	22,9	5,3	VA	VD (meio do septo)			X
8	14,2	21,0	6,9	TRN	AD (posterosseptal)			
9	15,2	20,3	5,0	TRN	AD (posterosseptal)			X
10	16,2	19,2	3,1	TRN	AD (posterosseptal)			
11	16,3	20,2	4,0	TRN	AD (posterosseptal)			
12	13,7	21,2	7,5	TRN	AD (posterosseptal)	X		
13	12,7	23,6	10,9	TA	AD (posterosseptal)	X		
14	13,5	19,7	6,2	TRN	AD (posterosseptal)			
15	10,9	18,0	7,1	TRN	AD (posterosseptal)			
16	10,3	20,5	10,2	TRN	AD (posterosseptal)			
17	17,2	29,1	11,9	TRN	AD (posterosseptal)			
18	15,3	20,0	4,8	TRN	AD (posterosseptal)			
19	10,5	18,3	7,8	VA	Parede lateral do AE			
20	18,3	23,9	5,6	VA	AD (anterosseptal)		X	

VA: via acessória; TRN: taquicardia por reentrada nodal; RMC: ressonância magnética cardíaca; TA: taquicardia atrial; AE: átrio esquerdo; AD: átrio direito; VD: ventrículo direito.

**Figura 2:**
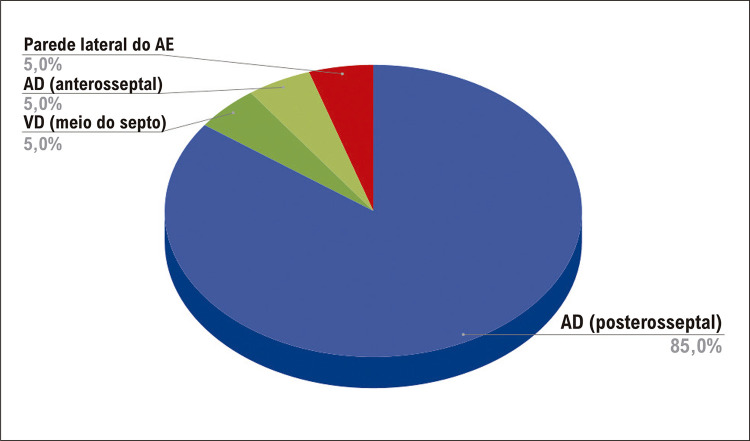
– Local de ablação dos 20 pacientes; AE: átrio esquerdo; AD: átrio direito; VD: ventrículo direito.

A localização das duas outras VAs foi lateral esquerda (n=1) e lateral direita (n=1). No paciente com VA lateral direita, condução decremental da VA de Mahaim foi observada. O mapeamento do átrio direito não mostrou potencial da VA e ablação no anel tricúspide a partir do átrio direito não foi bem-sucedido. A aplicação de RF foi realizada com sucesso na inserção ventricular da VA, na porção central do septal do ventrículo direito.

**Figura 3:**
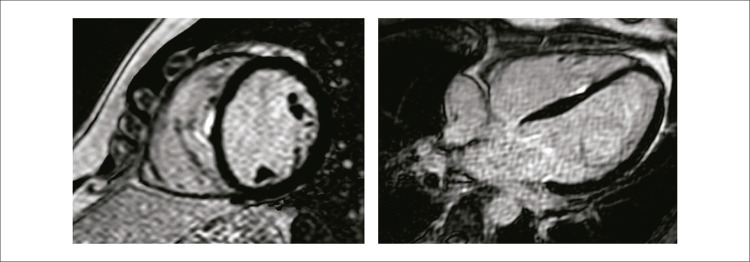
– Imagem de ressonância magnética da fibrose ventricular no paciente 7 (~0,6g).

A RMC e a TC foram realizadas em um tempo mediano de sete anos (Q1: 5, Q3: 10) após o procedimento de ablação. Três pacientes (15%) apresentaram fibrose miocárdica no ventrículo, visualizada por RMC com RTG, com um volume <0,6 cm ^3^ , predominantemente no segmento inferosseptal, e um padrão subendocárdico nos ventrículos direito e esquerdo correlacionado com os locais de ablação (posterosseptal). A porcentagem da área de fibrose foi inferior a 1% da massa do miocárdio do ventrículo esquerdo – 0,40%, 0,48% e 0,67% nesses pacientes ( Tabela 2 , Figura 3 ).

**Tabela 2:** – Quantificação da fibrose ventricular

Paciente	Quantificação da fibrose ventricular
4	0,46 mL ou cm ^3^ (460 mm ^3^ ) ~ 0,5g
7	0,57 mL ou cm ^3^ (570 mm ^3^ ) ~ 0,6g
9	0,54 mL ou cm ^3^ (540 mm ^3^ ) ~ 0,6g

RTG atrial foi observado em seis pacientes, a maioria localizado na porção posterosseptal e, em um caso, na parede posterior do átrio esquerdo.

Nenhum paciente apresentou lesões na artéria coronária na AngioTC, nem estenose ou calcificação.

## Discussão

A ablação com cateter tornou-se o tratamento padrão para arritmias sintomáticas e fatais, com uma alta taxa de sucesso, inclusive na população pediátrica. Embora sua baixa taxa de complicações esteja bem estabelecida em pacientes adultos, isso ainda é questionável em crianças. ^1^

As taxas de mortalidade em criança são baixas, aproximadamente 2/1000 casos no geral e 1/1000 casos em pacientes com coração com estrutura normal, ^2^ e pode ser ainda mais baixa, considerando técnicas e tecnologias recentes. Apesar da baixa mortalidade, há pouca evidência da evolução ou complicações em longo prazo e preocupações acerca do aumento das lesões da ablação foram relatadas. ^1^

Um estudo ^4^ experimental não mostrou alteração da dimensão da lesão ao longo do tempo em cães. Por outro lado, Saul et al. ^5^ conduziram um experimento com ovelhas jovens e observaram um aumento nas áreas de fibrose relacionada à aplicação de RF com o aumento do animal e do miocárdio, apesar do período de acompanhamento mais curto (8,5 ± 0,5 meses) quando comparado ao nosso. Contudo, não existem dados comparando a faixa etária entre diferentes espécies e possíveis diferenças na fisiologia.

O resfriamento convectivo é um efeito bem conhecido da perfusão coronária que previne complicações vasculares. Porém, se uma artéria estiver localizada dentro da área de ablação, sua eficiência pode ser comprometida. ^6^ Por outro lado, aplicações repetidas de RF para evitar o efeito de resfriamento pode levar ao dano vascular.

Paul et al. ^7^ demonstraram, em porcos jovens, o possível envolvimento da artéria coronária direita pelo espessamento significativo da camada íntima, relacionado à aplicação de RF na face atrial do anel da válvula tricúspide, observado 48 horas ou seis meses depois. ^8^ Em um estudo consecutivo envolvendo 212 pacientes, com idade mediana de 12 (0,3 – 20,4) anos, submetidos à angiografia coronária antes e 30 minutos após a ablação supraventricular com cateter, uma redução aguda no diâmetro luminal da artéria coronária foi encontrada em dois pacientes. Ambos os pacientes apresentaram uma VA posterosseptal. Em um dos casos, observou-se uma diminuição de 40% no diâmetro luminal da artéria coronária direita e, no outro, observou-se uma diminuição de 35% no diâmetro luminal da artéria circunflexa esquerda. Esses achados podem ser explicados por espasmo ou edema da artéria coronária, o que pode ser autolimitado, embora representem um dano arterial permanente relacionado à trombose, hiperplasia da camada íntima ou adventícia e necrose medial.

O RTG na RMC tornou-se uma ferramenta promissora na eletrofisiologia. A avaliação de lesões por RF pode potencialmente demonstrar a eficácia e a segurança desse tratamento, explicada pela coagulação grave e necrose de bandas de contração, com total perda da arquitetura celular e vascular nas lesões por RF, ^9 , 10^ mesmo se algumas das propriedades eletrofisiológicas possam ser revertidas após a fase aguda da aplicação da RF. ^11^

Na ocasião da RMC, a idade mediana dos pacientes era 20,5 anos, e a avaliação por imagem foi realizada 6,7 anos (mediana) após a ablação. Esse longo período após o procedimento sem RMC ou sinais de eventos adversos sugere que a técnica é segura nos pacientes com idade pediátrica. Além disso, a fibrose do miocárdio na RMC em pacientes após a ablação mostrou somente uma extensão limitada da lesão. Essa pequena quantidade de fibrose está provavelmente associada à lesão causada por ablação por RF e é necessária para o efeito terapêutico; porém, não provocou nenhum efeito negativo no desenvolvimento do miocárdio em nossa coorte.

Em nossos casos, a ablação foi realizada com cateteres não irrigados, mas, ao contrário do que era esperado, os cateteres com pontas irrigadas podem não resultar em lesões de volumes maiores em comparação a cateteres com pontas não irrigada na ablação de pacientes pediátricos. Isso pode ser explicado pelo fato do uso de que a potência aplicada é geralmente mais baixa, embora os cateteres irrigados possam resultar em melhores desfechos devido à maior precisão do mapeamento que eles possibilitam. ^10^

Observamos uma baixa taxa de fibrose atrial e ventricular, e ausência de disfunção ventricular, sete anos após a ablação por RF realizada durante meados da infância e adolescência.

Esses dados sugerem a hipótese de que a ablação por RF não aumenta as chances de arritmias ou disfunção ventricular esquerda durante o acompanhamento e seguimento dessas crianças.

Nesse cenário, a RMC é mais adequada na fase adulta, pois na maioria dos casos não há necessidade de sedação. Mais estudos são necessários para melhor avaliar pacientes mais jovens.

Embora estudos maiores com pacientes mais jovens sejam necessários, acreditamos que nossos dados reforça a segurança da ablação da taquicardia supraventricular por cateter de RF na população pediátrica, especialmente quanto à ausência de progressão da fibrose miocárdica e de lesões na artéria coronária após a ablação por RF nessa população.

### Limitações

A principal limitação de nosso estudo é o fato de ele haver sido conduzido com crianças em idade escolar e adolescentes, em que o impacto da expansão da fibrose seria menos esperada em comparação a ablações em neonatos, lactentes e pré-escolares.

Os pacientes não foram submetidos nem à tomografia nem a uma RMC antes da ablação para fins de comparação. No entanto, nos três pacientes em que o realce tardio ventricular (fibrose no miocárdio) foi observado na RMC, a área de fibrose sobrepôs-se aos locais de aplicação de RF.

Infelizmente, também tivemos um número limitado de pacientes e detalhes sobre o procedimento de ablação, tais como o número de aplicações e a potência mediana alcançada, não puderam ser recuperados. Mais estudos são necessários para corroborar o fato de que a ablação por RF é um procedimento seguro quando realizado em centros especializados.

## Conclusões

Nosso estudo demonstrou que o uso de RF no miocárdio em desenvolvimento pode de fato levar à fibrose, mas na minoria dos casos e em quantidade não significativa. No acompanhamento em longo prazo, nós não detectamos envolvimento coronariano e não observamos massa de fibrose significativa após o período de acompanhamento de 7±4 anos.
