# Supplementary material for: Progressão da Lesão Miocárdica após Ablação por Radiofrequência em Crianças em Idade Escolar
Source: Arq Bras Cardiol. 2024 Jan 24;121(1):e20220727. [Article in Portuguese] doi: 10.36660/abc.20220727 (PMC11098582; doi:10.36660/abc.20220727)
Supplement: Translation

# Translation: Myocardial Injury Progression after Radiofrequency Ablation in School-Age Children

**Authors:** Sissy Lara de Melo, Alberto Pereira Ferraz, Stephanie Ondracek Lemouche, Marcela Santana Devido, Gabriela Liberato de Sousa, Carlos E. Rochitte, Cristiano Faria Pisani, Denise Tessariol Hachul, Mauricio Scanavacca

**Affiliations:** 1 Brazil Instituto do Coração do Hospital das Clínicas da Faculdade de Medicina da Universidade de São Paulo, São Paulo, SP – Brazil

**Keywords:** Catheter Ablation, Myocardial Contusions, Child, Safety

## Abstract

**Background:**

The past decades have seen the rapid development of the invasive treatment of arrhythmias by catheter ablation procedures. Despite its safety and efficacy being well-established in adults, to date there has been little data in pediatric scenarios. One of the main concerns is the possible expansion of the ablation procedure scar in this population and its consequences over the years.

**Objectives:**

This study aimed to analyze the risk of myocardial injury progression after radiofrequency catheter ablation in pediatric patients.

**Methods:**

This is a retrospective study of 20 pediatric patients with previous ablation for treatment of supraventricular arrhythmia that underwent cardiac magnetic resonance and coronary angiography for evaluation of myocardial fibrosis and the integrity of the coronary arteries during follow-up.

**Results:**

The median age at ablation procedure was 15.1 years (Q1 12.9, Q3 16.6) and 21 years (Q1 20, Q3 23) when the cardiac magnetic resonance was performed. Fourteen of them were women. Nodal reentry tachycardia and Wolf-Parkinson-White Syndrome were the main diagnosis (19 patients), with one patient with atrial tachycardia. Three patients had ventricular myocardial fibrosis, but with a volume < 0.6 cm ^3^ . None of them developed ventricular dysfunction and no patient had coronary lesions on angiography.

**Conclusion:**

Radiofrequency catheter ablation did not show to increase the risk of myocardial injury progression or coronary artery lesions.

**Central Illustration:** : Main results of radiofrequency ablation pediatric cases evaluated by cardiac magnetic resonance imaging and computed tomography coronary angiography late after the procedure

## Introduction

The effectiveness and safety of treating arrhythmias using radiofrequency (RF) ablation is well established in the current medical practice. ^1 , 2^ In the pediatric population above five years of age and weight greater than 15Kg, it has become a routine procedure over the past few years. ^3^ But there are few studies and lack of evidence of the possible impact of RF application on the development of the myocardium in school-age children and adolescents. Most current assumptions are inferences from animal studies. ^4 , 5^ Main concerns include the possible involvement of coronary arteries and progression of myocardial fibrosis during adulthood. There is little data in the literature that allows us to estimate the behavior of these injuries during the growth of the myocardium, whether they tend to increase or not over the years and what would be the consequences on the child’s cardiac development.

Most studies in the literature demonstrated the consequences in the short term, from hours to a few months after the RF application. Our study aimed to identify the presence of coronary artery lesions and extent of myocardial fibrosis after application of RF in pediatric patients during a long-term follow-up.

## Methods

All the 187 patients that underwent RF catheter ablation in a tertiary cardiology center at the age of 15 years or younger and were 18 years old or older when the study was started (2015) were contacted and invited for participation. After the invitation, 20 patients consented the participation and informed consent was obtained from the parents or legally authorized representative. Research protocols were reviewed and approved by protocol number SDC 3776/12/032.

The RF catheter ablation procedures were performed between July 2002 and November 2012. Through puncture of the right and / or left femoral vein and under general anesthesia, multipolar electrode catheters were introduced using fluoroscopic guidance for electrophysiological mapping and application of RF pulses. RF energy was delivered using 4mm tip catheters in most cases, except for one patient with Mahaim tachycardia in whom an 8mm tip catheter was necessary, after failure of ablation with a 4mm tip catheter. The temperature was set at 60ºC and power at 50W for all cases.

For myocardial fibrosis evaluation, a cardiac magnetic resonance (CMR) was performed, and coronary angiography (Cor) by computed tomography (CT) (CorCTA) was used for coronary artery integrity investigation. All examinations were conducted in a Philips Achieva 1.5T MRI scanner (Netherlands). Ventricular function and volumes were assessed by cine resonance, using the sequence, and subsequently obtained by the Simpson’s method with images on the short axis of the heart. Myocardial fibrosis was evaluated using the myocardial delayed enhancement technique, also known as late gadolinium enhancement (LGE), in 2D and 3D acquisitions, assessing the atria and ventricles. All analyzes were performed using the CVi42 software (Circle CVi, Calgary, Canada). CorCTA was performed on a 320 x 0.5mm detector (Aquilion ONE, Canon Medical Systems, Otawara, Japan). The protocol included the acquisition of non-contrasted images for calcium score and the acquisition of CorCTA with iodinated contrast injection (50-70mL) and prospective electrocardiographic synchronization for evaluation of coronary artery lumen.

### Statistical analysis

A descriptive analysis was performed. The numerical data are presented as means and standard deviations or medians and interquartile ranges, according to normality of the data. Categorized data are presented as absolute frequency and percentages. Data were tested for distribution normality using the Shapiro-Wilk test of normality. A P-value of <0.05 was considered statistically significant. The IBM-SPSS for Windows version 25.0 software was used for the analyzes and the Microsoft Excel 2013 software was used to tabulate the data.

## Results

We performed CT and CRM in 20 patients, most were female (70%), median age of 15.1 (Q1: 12.9; Q3: 16.67) years old at the time of ablation and 20.8 (Q1: 20 and Q3: 23.5) years-old at the time of evaluation. The median time from ablation to evaluation was 6.7 (Q1: 5.37; Q3: 9.12) years. Mean body weight, height and body mass index were 65.7±9Kg, 167.6±7.5cm and 23.4±2.3 Kg/m ^2^ , respectively. The ejection fraction was 61±8% and only one patient diagnosed with atrial tachycardia (AT) had structural heart disease: an atrial septal defect, surgically corrected by atrioseptoplasty with patch placement.

Eighteen patients underwent ablation in the right septal region: 13 with atrioventricular nodal reentry tachycardia and four with Wolff-Parkinson-White Syndrome. The locations of these accessory pathways (AP) were anteroseptal (n=1) e posteroseptal (n=3). One patient had AT located in the posteroseptal region ( Table 1 , Figure 1 and 2).

**Table 1:** – Characteristics of patients and procedures performed

Patient	Age of ablation (years)	Age at CMR (years)	Time of CMR after ablation (years)	Diagnosis	Ablation site	RA Fibrosis	LA Fibrosis	Ventricular fibrosis
1	12.3	20.6	8.3	AVNRT	Posteroseptal RA			
2	14.3	20.5	6.2	AP	Posteroseptal RA			
3	17.2	23.4	6.2	AVNRT	Posteroseptal RA	X	X	
4	16.8	26.2	9.4	AP	Posteroseptal RA		X	X
5	16.3	27.5	11.2	AVNRT	Posteroseptal RA		X	
6	15.0	21.5	6.5	AP	Posteroseptal RA			
7	17.6	22.9	5.3	AP	Mindseptal of RV			X
8	14.2	21.0	6.9	AVNRT	Posteroseptal RA			
9	15.2	20.3	5.0	AVNRT	Posteroseptal RA			X
10	16.2	19.2	3.1	AVNRT	Posteroseptal RA			
11	16.3	20.2	4.0	AVNRT	Posteroseptal RA			
12	13.7	21.2	7.5	AVNRT	Posteroseptal RA	X		
13	12.7	23.6	10.9	AT	Posteroseptal RA	X		
14	13.5	19.7	6.2	AVNRT	Posteroseptal RA			
15	10.9	18.0	7.1	AVNRT	Posteroseptal RA			
16	10.3	20.5	10.2	AVNRT	Posteroseptal RA			
17	17.2	29.1	11.9	AVNRT	Posteroseptal RA			
18	15.3	20.0	4.8	AVNRT	Posteroseptal RA			
19	10.5	18.3	7.8	AP	Lateral wall of LA			
20	18.3	23.9	5.6	AP	Anteroseptal RA		X	

AP: accessory pathway; AT: atrial tachycardia; AVNRT: atrioventricular nodal reentry tachycardia; CMR: cardiac magnetic resonance; LA: left atrium; RA: right atrium; RV: right ventricle.

**Figure 1:** – Electrophysiological diagnosis; AP: accessory pathway; AT: atrial tachycardia; AVNRT: atrioventricular nodal reentry tachycardia.

**Figure 2:** – Ablation site of the 20 patients.

The location of the two others AP was left lateral (n=1) and right lateral (n=1). In the patient with right lateral AP, decremental conduction of the Mahaim AP was observed. Right atrium mapping did not show potential of the AP and ablation in the tricuspid ring from the right atrium was unsuccessful. RF applications were successfully performed in the ventricular insertion of the AP, at the midseptal portion of the right ventricle.

The CMR and CT were performed in a median time of seven years (Q1: 5, Q3: 10) after the RF catheter ablation procedure. Three patients (15%) had ventricular myocardial fibrosis visualized by LGE in CMR, with a volume <0.6 cm, ^3^ predominantly in the inferoseptal segment, with a subendocardial pattern in the left and right ventricles correlated with the ablation sites (posteroseptal). The percentage of the area of fibrosis was less than 1% of the myocardial left ventricular mass – 0.40%, 0.48% and 0.67% in these patients ( Table 2 , Figure 3 ).

**Table 2:** – Quantification of ventricular fibrosis

Patient	Quantification of ventricular fibrosis
4	0,46 mL or cm ^3^ (460 mm ^3^ ) ~ 0,5g
7	0,57 mL or cm ^3^ (570 mm ^3^ ) ~ 0,6g
9	0,54 mL or cm ^3^ (540 mm ^3^ ) ~ 0,6g

**Figure 3:** – Magnetic resonance image of ventricular fibrosis in patient 7 (~0.6g).

Atrial LGE was observed in six patients, mostly located in the posteroseptal portion and, in one case, in the posterior wall of the left atrium.

No patient had coronary artery lesions by CorCTA, either stenosis or calcification.

## Discussion

Catheter ablation has become the standard treatment for symptomatic and life-threatening arrhythmias, with a high procedural success rate, also in the pediatric population. Its low rate of complications is well established in adult patients, but it is still questionable in pediatrics. ^1^

Mortality rates in children are low, approximately 2/1000 cases overall and 1/1000 cases for patients with structurally normal hearts ^2^ and can be even lower, considering recent techniques and technologies. Despite its low mortality, there is a lack of evidence of long-term evolution or complications and concerns about the enlargement of ablation lesions has been postulated. ^1^

An experimental study ^4^ showed no lesion dimension change over time in dogs. Differently, Saul et al. ^5^ carried out an experiment with young sheeps and observed an increase in the areas of fibrosis related to RF application along the growth of the animal and the myocardium, although their follow-up period was shorter (8.5 ± 0.5 months), when compared to ours. Nevertheless, there are no data comparing age range between different species and possible differences in their physiology.

Convective cooling is a well-known effect of coronary perfusion that prevents vascular complications. But, if an artery is located inside the ablative target area, the efficiency of the ablation may be compromised. ^6^ On the other hand, repeated RF applications to avoid the cooling effect can lead to vascular damage.

Paul et al. ^7^ demonstrated, in young pigs, the possible involvement of the right coronary artery by significant intimal thickening related to RF application in the atrial face of the tricuspid valve ring, observed 48h or 6 months later.~ ^8^ In a consecutive study involving 212 patients, with a median age of 12 (0.3 - 20.4) years old, who underwent coronary angiography before and 30 minutes after supraventricular catheter ablation, an acute reduction in the luminal diameter of the coronary artery was found in two patients. Both patients had a posteroseptal AP. A 40% decrease in the luminal diameter of the distal right coronary artery was observed in one case and a 35% decrease in the luminal diameter of left circumflex coronary artery in the other. These findings may be explained by coronary spasm or edema, which might be self-limited, although they can represent permanent arterial damage related to thrombosis, intimal or adventitial hyperplasia and medial necrosis. ^8^

LGE in CMR has become a promising tool in electrophysiology scenarios. Assessment of RF lesions can potentially demonstrate efficacy and security of this treatment, explained by the severe coagulation and contraction band necrosis with complete loss of cellular and vascular architecture in RF lesions, ^9 , 10^ even though some of the electrophysiologic properties can be reversed after the acute phase of RF energy delivery. ^11^

At the time of our CMR study, patients had a median age of 20.5 years old, and imaging evaluation was performed 6.7 years (median) after ablation. This long period after the procedure without CMR or clinical signs of adverse events suggest that the technique is safe in the pediatric age group. Additionally, myocardial fibrosis on CMR in patients after ablation showed only limited lesion extension. This small amount of myocardial fibrosis is probably related to the lesion caused by RF ablation and necessary for the therapeutic effect but did not provoke any negative effect on myocardial development in this cohort.

In our cases, ablation was performed with non-irrigated catheters, but contrary to what should be expected, irrigated-tip catheters may not result in larger lesion volume than non-irrigated-tip catheters in pediatrics ablation. The reason for that may be the fact that the power is usually decreased, although irrigated catheters may result in better successful outcomes due to the more precise mapping they can enable. ^10^

We observed only a small rate of ventricular and atrial fibrosis and no ventricular dysfunction, seven years after RF ablation, performed during middle childhood and adolescence.

These data raise the hypothesis that RF ablation does not increase the chances of arrhythmias or left ventricular dysfunction during follow-up and development of these children.

In this scenario, CMR is best suited in adulthood, in whom in most cases, there is no longer need of sedation. Further studies are necessary to better evaluate younger aged patients.

Although larger studies in younger patients are necessary, we believe that our data reassures the safety of RF ablation of supraventricular tachycardia in the pediatric population, especially regarding the absence of myocardial fibrosis progression and coronary artery lesions after RF application in this population in long term follow-up.

### Limitations

The main limitation of our study is that it was carried out in school-age children and adolescents in whom the impact of fibrosis expansion would be less expected when compared to ablations in neonates, infants and preschoolers.

The patients did not undergo a pre-ablation tomography or CMR images for comparison. However, in the three patients in whom late ventricular enhancement (myocardial fibrosis) was observed by CMR, the area of fibrosis overlapped the sites of RF application.

Unfortunately, we also had a limited number of patients and details of the ablation procedure such as number of applications and median power reached could not be retrieved. Further studies are necessary to reinforce the fact that the RF ablation is safe when performed in specialized centers.

## Conclusions

Our study demonstrated that the use of RF in the developing myocardium might indeed lead to fibrosis, but in the minority of cases and in no significant quantity. In the long-term follow-up, we did not detect coronary involvement and there was no significant fibrosis mass after the follow-up period of 7±4 years.
